# Ethyl 8-chloro-1-cyclo­propyl-6,7-difluoro-4-oxo-1,4-dihydro­quinoline-3-carboxyl­ate

**DOI:** 10.1107/S160053681104205X

**Published:** 2011-10-22

**Authors:** Hong-shun Sun, Long Jiang, Yu-Long Li, Xin-hua Lu, Hong Xu

**Affiliations:** a Department of Applied Chemistry, Nanjing College of Chemical Technology, Geguan Road No. 265 Nanjing, Nanjing 210048, People’s Republic of China; b R&D Center, Jiangsu Yabang Pharmaceutical Group, Liangchang Road East No. 6 Jingtan, Changzhou 213200, People’s Republic of China; c Department of Chemical Engineering, Nanjing College of Chemical Technology, Geguan Road No. 265 Nanjing, Nanjing 210048, People’s Republic of China

## Abstract

In the mol­ecule of the title compound, C_15_H_12_ClF_2_NO_3_, the quinoline ring system is not planar, the dihedral angle between the pyridine and benzene rings being 3.55 (8)°. In the crystal, inter­molecular C—H⋯O hydrogen bonds link the mol­ecules into layers parallel to (101).

## Related literature

For the anti­bacterial activity of quinolone derivatives, see: Fujita & Chiba (1998 ▶). For a related structure, see: Wang *et al.* (2008 ▶).
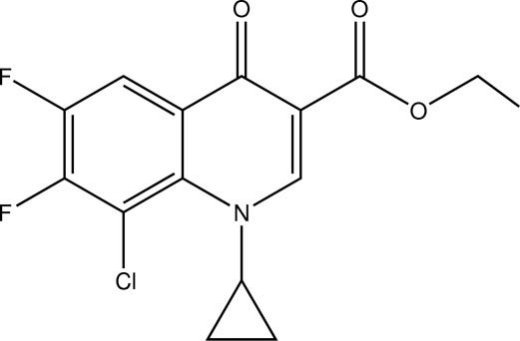


## Experimental

### 

#### Crystal data

C_15_H_12_ClF_2_NO_3_
*M* *_r_* = 327.71
Monoclinic, 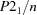

*a* = 11.336 (2) Å
*b* = 7.7440 (15) Å
*c* = 16.157 (3) Å
β = 95.40 (3)°
*V* = 1412.1 (5) Å^3^
*Z* = 4
Mo *K*α radiation
μ = 0.31 mm^−1^
*T* = 293 K
0.30 × 0.20 × 0.10 mm

#### Data collection

Enraf–Nonius CAD-4 diffractometer
Absorption correction: ψ scan (North *et al.*, 1968 ▶) *T*_min_ = 0.914, *T*_max_ = 0.970
2741 measured reflections
2604 independent reflections
1728 reflections with *I* > 2σ(*I*)
*R*_int_ = 0.025
3 standard reflections every 200 reflections  intensity decay: 1%

#### Refinement

*R*[*F* ^2^ > 2σ(*F* ^2^)] = 0.053
*wR*(*F* ^2^) = 0.158
*S* = 1.00
2604 reflections
200 parameters
H-atom parameters constrained
Δρ_max_ = 0.21 e Å^−3^
Δρ_min_ = −0.25 e Å^−3^

### 

Data collection: *CAD-4 EXPRESS* (Enraf–Nonius, 1994 ▶); cell refinement: *CAD-4 EXPRESS*; data reduction: *XCAD4* (Harms & Wocadlo, 1995 ▶); program(s) used to solve structure: *SHELXS97* (Sheldrick, 2008 ▶); program(s) used to refine structure: *SHELXL97* (Sheldrick, 2008 ▶); molecular graphics: *SHELXTL* (Sheldrick, 2008 ▶); software used to prepare material for publication: *SHELXTL*.

## Supplementary Material

Crystal structure: contains datablock(s) I, global. DOI: 10.1107/S160053681104205X/rz2646sup1.cif

Structure factors: contains datablock(s) I. DOI: 10.1107/S160053681104205X/rz2646Isup2.hkl

Supplementary material file. DOI: 10.1107/S160053681104205X/rz2646Isup3.cml

Additional supplementary materials:  crystallographic information; 3D view; checkCIF report

## Figures and Tables

**Table 1:** Hydrogen-bond geometry (Å, °)

*D*—H⋯*A*	*D*—H	H⋯*A*	*D*⋯*A*	*D*—H⋯*A*
C11—H11*A*⋯O1^i^	0.97	2.55	3.240 (4)	128
C11—H11*B*⋯O1^ii^	0.97	2.54	3.491 (4)	167

Symmetry codes: (i) 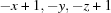; (ii) 
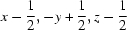
.

**Fig. 1.**
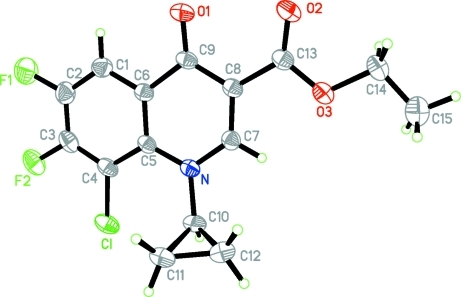
The molecular structure of the title compound, with displacement ellipsoids drawn at the 50% probability level.

## supplementary crystallographic information

### Comment

Quinolone antibacterials were found several decades ago, and some excellent antibacterials have been developed and used widely (Fujita & Chiba, 1998). An interest in the search of more potent antibacterial agents led us to design and synthesize a new type of quinoline derivative. The title compound is one of the key intermediates and we report here its crystal structure.

The quinoline ring system is not planar, the dihedral angle between the pyridine and benzene rings being 3.55 (8)°. The dihedral angle between the three-membered ring and the quinoline ring system is 80.5 (5)°. Bond lengths and angles agree well with those observed in the strictly related compound ethyl 1-cyclopropyl-6,7-difluoro-8-methoxy-4-oxo-1,4-dihydroquinoline-3-carboxylate reported recently (Wang *et al.*, 2008). In the crystal structure, intermolecular C—H···O hydrogen bonds link molecules into layers parallel to the (101) plane.

### Experimental

A solution of 3-cyclopropylamino-2-(2,4,5-trifluoro-3-chlorobenzoyl)acrylic acid ethyl ester (26.1 g, 0.075 mol) in DMF (110 ml) was treated with K_2_CO_3_ (22 g, 0.16 mol) and then heated to 50°C with stirring for 1 h. The resulting precipitate was filtered, washed with a mixture of ice and water, and dried to give 22 g of the title compound (yield 90%). Crystals of the title compound suitable for X-ray diffraction analysis were obtained by slow evaporation of an acetone solution.

### Refinement

All H atoms were positioned geometrically and refined using a riding model with C—H = 0.93–0.98 Å and *U*_iso_(H) = 1.2 or 1.5 *U*_eq_(C). A rotating-group model was applied for the methyl groups.

### Crystal data

**Table:**

C_15_H_12_ClF_2_NO_3_	*F*(000) = 672
*M**_r_* = 327.71	*D*_x_ = 1.541 Mg m^−^^3^
Monoclinic, *P*2_1_/*n*	Mo *K*α radiation, λ = 0.71073 Å
Hall symbol: -P 2yn	Cell parameters from 25 reflections
*a* = 11.336 (2) Å	θ = 9–13°
*b* = 7.7440 (15) Å	µ = 0.31 mm^−^^1^
*c* = 16.157 (3) Å	*T* = 293 K
β = 95.40 (3)°	Block, colourless
*V* = 1412.1 (5) Å^3^	0.30 × 0.20 × 0.10 mm
*Z* = 4	

### Data collection

**Table:**

Enraf–Nonius CAD-4 diffractometer	1728 reflections with *I* > 2σ(*I*)
Radiation source: fine-focus sealed tube	*R*_int_ = 0.025
graphite	θ_max_ = 25.4°, θ_min_ = 2.1°
ω/2θ scans	*h* = 0→13
Absorption correction: ψ scan (North *et al.*, 1968)	*k* = 0→9
*T*_min_ = 0.914, *T*_max_ = 0.970	*l* = −19→19
2741 measured reflections	3 standard reflections every 200 reflections
2604 independent reflections	intensity decay: 1%

### Refinement

**Table:**

Refinement on *F*^2^	Secondary atom site location: difference Fourier map
Least-squares matrix: full	Hydrogen site location: inferred from neighbouring sites
*R*[*F*^2^ > 2σ(*F*^2^)] = 0.053	H-atom parameters constrained
*wR*(*F*^2^) = 0.158	*w* = 1/[σ^2^(*F*_o_^2^) + (0.094*P*)^2^] where *P* = (*F*_o_^2^ + 2*F*_c_^2^)/3
*S* = 1.00	(Δ/σ)_max_ < 0.001
2604 reflections	Δρ_max_ = 0.21 e Å^−^^3^
200 parameters	Δρ_min_ = −0.25 e Å^−^^3^
0 restraints	Extinction correction: *SHELXL97* (Sheldrick, 2008), Fc^*^=kFc[1+0.001xFc^2^λ^3^/sin(2θ)]^-1/4^
Primary atom site location: structure-invariant direct methods	Extinction coefficient: 0.022 (3)

### Special details

**Table:**

Geometry. All e.s.d.'s (except the e.s.d. in the dihedral angle between two l.s. planes) are estimated using the full covariance matrix. The cell e.s.d.'s are taken into account individually in the estimation of e.s.d.'s in distances, angles and torsion angles; correlations between e.s.d.'s in cell parameters are only used when they are defined by crystal symmetry. An approximate (isotropic) treatment of cell e.s.d.'s is used for estimating e.s.d.'s involving l.s. planes.
Refinement. Refinement of *F*^2^ against ALL reflections. The weighted *R*-factor *wR* and goodness of fit *S* are based on *F*^2^, conventional *R*-factors *R* are based on *F*, with *F* set to zero for negative *F*^2^. The threshold expression of *F*^2^ > σ(*F*^2^) is used only for calculating *R*-factors(gt) *etc*. and is not relevant to the choice of reflections for refinement. *R*-factors based on *F*^2^ are statistically about twice as large as those based on *F*, and *R*- factors based on ALL data will be even larger.

### Fractional atomic coordinates and isotropic or equivalent isotropic displacement parameters (Å^2^)

**Table:**

	*x*	*y*	*z*	*U*_iso_*/*U*_eq_	
Cl	0.19669 (7)	0.51225 (11)	0.40024 (6)	0.0668 (4)	
N	0.44922 (19)	0.3170 (3)	0.40949 (14)	0.0419 (6)	
O1	0.59434 (19)	0.1055 (3)	0.62827 (13)	0.0642 (7)	
C1	0.3696 (3)	0.2253 (4)	0.62119 (19)	0.0544 (8)	
H1A	0.4037	0.1728	0.6694	0.065*	
F1	0.19320 (19)	0.2766 (4)	0.68412 (14)	0.0983 (8)	
O2	0.79506 (19)	0.0443 (3)	0.54403 (15)	0.0651 (7)	
F2	0.09874 (16)	0.4358 (3)	0.54885 (14)	0.0800 (7)	
C2	0.2582 (3)	0.2886 (5)	0.6180 (2)	0.0631 (9)	
O3	0.79232 (18)	0.1827 (3)	0.42263 (14)	0.0627 (6)	
C3	0.2086 (3)	0.3706 (4)	0.5476 (2)	0.0570 (9)	
C4	0.2681 (3)	0.3882 (4)	0.47802 (19)	0.0485 (8)	
C5	0.3828 (2)	0.3153 (3)	0.47746 (17)	0.0399 (7)	
C6	0.4329 (2)	0.2392 (4)	0.55187 (17)	0.0420 (7)	
C7	0.5626 (2)	0.2603 (4)	0.41917 (17)	0.0436 (7)	
H7A	0.6060	0.2690	0.3733	0.052*	
C8	0.6190 (2)	0.1915 (4)	0.48987 (17)	0.0421 (7)	
C9	0.5555 (2)	0.1703 (4)	0.56176 (17)	0.0440 (7)	
C10	0.4003 (3)	0.3598 (4)	0.32426 (17)	0.0499 (8)	
H10A	0.3941	0.4831	0.3109	0.060*	
C11	0.3070 (3)	0.2476 (4)	0.28256 (18)	0.0569 (9)	
H11A	0.2816	0.1486	0.3131	0.068*	
H11B	0.2450	0.3023	0.2462	0.068*	
C12	0.4275 (3)	0.2441 (5)	0.25528 (18)	0.0630 (9)	
H12A	0.4393	0.2967	0.2022	0.076*	
H12B	0.4759	0.1431	0.2692	0.076*	
C13	0.7432 (3)	0.1294 (4)	0.49057 (19)	0.0475 (7)	
C14	0.9161 (3)	0.1308 (6)	0.4192 (2)	0.0697 (10)	
H14A	0.9241	0.0067	0.4261	0.084*	
H14B	0.9663	0.1869	0.4632	0.084*	
C15	0.9506 (4)	0.1833 (6)	0.3377 (2)	0.0891 (13)	
H15A	1.0316	0.1516	0.3332	0.134*	
H15B	0.9421	0.3062	0.3317	0.134*	
H15C	0.9005	0.1266	0.2947	0.134*	

### Atomic displacement parameters (Å^2^)

**Table:**

	*U*^11^	*U*^22^	*U*^33^	*U*^12^	*U*^13^	*U*^23^
Cl	0.0592 (5)	0.0603 (6)	0.0764 (6)	0.0187 (4)	−0.0166 (4)	−0.0001 (4)
N	0.0404 (13)	0.0399 (13)	0.0437 (13)	−0.0013 (10)	−0.0054 (10)	0.0007 (10)
O1	0.0579 (13)	0.0809 (17)	0.0511 (13)	0.0097 (12)	−0.0089 (10)	0.0147 (12)
C1	0.056 (2)	0.058 (2)	0.0477 (17)	−0.0018 (16)	0.0012 (15)	0.0002 (15)
F1	0.0732 (15)	0.146 (2)	0.0798 (14)	0.0125 (15)	0.0296 (12)	0.0032 (15)
O2	0.0507 (13)	0.0699 (16)	0.0727 (15)	0.0149 (11)	−0.0035 (12)	0.0137 (13)
F2	0.0461 (11)	0.0932 (16)	0.1010 (16)	0.0177 (11)	0.0077 (11)	−0.0139 (13)
C2	0.055 (2)	0.078 (2)	0.058 (2)	−0.0008 (18)	0.0145 (17)	−0.0068 (18)
O3	0.0421 (12)	0.0806 (17)	0.0646 (14)	0.0079 (11)	0.0012 (10)	0.0107 (13)
C3	0.0412 (17)	0.060 (2)	0.069 (2)	0.0050 (15)	0.0009 (16)	−0.0149 (18)
C4	0.0454 (17)	0.0388 (16)	0.0585 (19)	0.0015 (13)	−0.0102 (15)	−0.0070 (14)
C5	0.0364 (14)	0.0321 (14)	0.0493 (16)	−0.0045 (12)	−0.0057 (12)	−0.0023 (12)
C6	0.0423 (16)	0.0381 (15)	0.0440 (15)	−0.0032 (12)	−0.0039 (13)	−0.0028 (13)
C7	0.0408 (16)	0.0427 (16)	0.0470 (16)	−0.0042 (13)	0.0024 (13)	−0.0002 (14)
C8	0.0396 (15)	0.0378 (15)	0.0471 (16)	−0.0035 (12)	−0.0043 (13)	−0.0024 (13)
C9	0.0445 (16)	0.0395 (16)	0.0452 (17)	−0.0026 (13)	−0.0110 (13)	−0.0020 (14)
C10	0.0549 (18)	0.0459 (17)	0.0460 (17)	0.0000 (14)	−0.0102 (14)	0.0065 (14)
C11	0.055 (2)	0.061 (2)	0.0516 (17)	0.0007 (16)	−0.0129 (15)	0.0014 (16)
C12	0.069 (2)	0.076 (2)	0.0430 (17)	0.0012 (19)	0.0009 (16)	0.0032 (17)
C13	0.0442 (16)	0.0441 (17)	0.0526 (18)	−0.0020 (14)	−0.0044 (14)	−0.0023 (15)
C14	0.0433 (18)	0.087 (3)	0.079 (2)	0.0094 (18)	0.0044 (17)	0.005 (2)
C15	0.072 (3)	0.106 (3)	0.092 (3)	0.008 (2)	0.022 (2)	0.004 (3)

### Geometric parameters (Å, °)

**Table:**

Cl—C4	1.723 (3)	C7—C8	1.364 (4)
N—C7	1.353 (3)	C7—H7A	0.9300
N—C5	1.389 (4)	C8—C9	1.432 (4)
N—C10	1.473 (3)	C8—C13	1.486 (4)
O1—C9	1.229 (3)	C10—C11	1.481 (4)
C1—C2	1.351 (5)	C10—C12	1.485 (4)
C1—C6	1.390 (4)	C10—H10A	0.9800
C1—H1A	0.9300	C11—C12	1.475 (5)
F1—C2	1.357 (4)	C11—H11A	0.9700
O2—C13	1.196 (3)	C11—H11B	0.9700
F2—C3	1.346 (4)	C12—H12A	0.9700
C2—C3	1.375 (5)	C12—H12B	0.9700
O3—C13	1.342 (4)	C14—C15	1.467 (5)
O3—C14	1.466 (4)	C14—H14A	0.9700
C3—C4	1.371 (4)	C14—H14B	0.9700
C4—C5	1.418 (4)	C15—H15A	0.9600
C5—C6	1.410 (4)	C15—H15B	0.9600
C6—C9	1.483 (4)	C15—H15C	0.9600
			
C7—N—C5	119.0 (2)	N—C10—C12	118.7 (3)
C7—N—C10	116.8 (2)	C11—C10—C12	59.6 (2)
C5—N—C10	123.8 (2)	N—C10—H10A	116.0
C2—C1—C6	119.5 (3)	C11—C10—H10A	116.0
C2—C1—H1A	120.3	C12—C10—H10A	116.0
C6—C1—H1A	120.3	C12—C11—C10	60.3 (2)
C1—C2—F1	121.3 (3)	C12—C11—H11A	117.7
C1—C2—C3	120.5 (3)	C10—C11—H11A	117.7
F1—C2—C3	118.2 (3)	C12—C11—H11B	117.7
C13—O3—C14	114.8 (2)	C10—C11—H11B	117.7
F2—C3—C4	120.2 (3)	H11A—C11—H11B	114.9
F2—C3—C2	117.9 (3)	C11—C12—C10	60.1 (2)
C4—C3—C2	121.9 (3)	C11—C12—H12A	117.8
C3—C4—C5	119.2 (3)	C10—C12—H12A	117.8
C3—C4—Cl	114.8 (2)	C11—C12—H12B	117.8
C5—C4—Cl	125.9 (3)	C10—C12—H12B	117.8
N—C5—C6	118.3 (2)	H12A—C12—H12B	114.9
N—C5—C4	124.5 (3)	O2—C13—O3	123.1 (3)
C6—C5—C4	117.2 (3)	O2—C13—C8	125.8 (3)
C1—C6—C5	121.5 (3)	O3—C13—C8	111.1 (3)
C1—C6—C9	115.8 (3)	O3—C14—C15	107.1 (3)
C5—C6—C9	122.7 (3)	O3—C14—H14A	110.3
N—C7—C8	126.1 (3)	C15—C14—H14A	110.3
N—C7—H7A	116.9	O3—C14—H14B	110.3
C8—C7—H7A	116.9	C15—C14—H14B	110.3
C7—C8—C9	119.4 (3)	H14A—C14—H14B	108.5
C7—C8—C13	120.1 (3)	C14—C15—H15A	109.5
C9—C8—C13	120.3 (3)	C14—C15—H15B	109.5
O1—C9—C8	126.1 (3)	H15A—C15—H15B	109.5
O1—C9—C6	119.7 (3)	C14—C15—H15C	109.5
C8—C9—C6	114.2 (2)	H15A—C15—H15C	109.5
N—C10—C11	118.9 (3)	H15B—C15—H15C	109.5
			
C6—C1—C2—F1	179.6 (3)	C10—N—C7—C8	−170.2 (3)
C6—C1—C2—C3	−1.8 (5)	N—C7—C8—C9	1.7 (4)
C1—C2—C3—F2	−178.0 (3)	N—C7—C8—C13	178.1 (3)
F1—C2—C3—F2	0.7 (5)	C7—C8—C9—O1	177.6 (3)
C1—C2—C3—C4	1.4 (5)	C13—C8—C9—O1	1.3 (5)
F1—C2—C3—C4	−179.9 (3)	C7—C8—C9—C6	−3.9 (4)
F2—C3—C4—C5	−178.7 (3)	C13—C8—C9—C6	179.8 (2)
C2—C3—C4—C5	2.0 (5)	C1—C6—C9—O1	0.3 (4)
F2—C3—C4—Cl	5.2 (4)	C5—C6—C9—O1	179.9 (3)
C2—C3—C4—Cl	−174.1 (3)	C1—C6—C9—C8	−178.3 (2)
C7—N—C5—C6	−5.9 (4)	C5—C6—C9—C8	1.3 (4)
C10—N—C5—C6	167.3 (2)	C7—N—C10—C11	110.6 (3)
C7—N—C5—C4	172.8 (3)	C5—N—C10—C11	−62.8 (4)
C10—N—C5—C4	−13.9 (4)	C7—N—C10—C12	41.4 (4)
C3—C4—C5—N	176.6 (3)	C5—N—C10—C12	−132.0 (3)
Cl—C4—C5—N	−7.8 (4)	N—C10—C11—C12	−108.2 (3)
C3—C4—C5—C6	−4.7 (4)	N—C10—C12—C11	108.5 (3)
Cl—C4—C5—C6	170.9 (2)	C14—O3—C13—O2	−0.9 (5)
C2—C1—C6—C5	−1.1 (5)	C14—O3—C13—C8	178.5 (3)
C2—C1—C6—C9	178.4 (3)	C7—C8—C13—O2	−168.0 (3)
N—C5—C6—C1	−176.8 (3)	C9—C8—C13—O2	8.3 (5)
C4—C5—C6—C1	4.3 (4)	C7—C8—C13—O3	12.5 (4)
N—C5—C6—C9	3.6 (4)	C9—C8—C13—O3	−171.2 (2)
C4—C5—C6—C9	−175.2 (3)	C13—O3—C14—C15	174.0 (3)
C5—N—C7—C8	3.5 (4)		

### Hydrogen-bond geometry (Å, °)

**Table:**

*D*—H···*A*	*D*—H	H···*A*	*D*···*A*	*D*—H···*A*
C11—H11A···O1^i^	0.97	2.55	3.240 (4)	128.
C11—H11B···O1^ii^	0.97	2.54	3.491 (4)	167.

Symmetry codes: (i) −*x*+1, −*y*, −*z*+1; (ii) *x*−1/2, −*y*+1/2, *z*−1/2.
